# The Chongqing Adolescent Twin Study: An Integrative Multimodal Brain Imaging and Non-imaging Dataset

**DOI:** 10.1038/s41597-025-05449-z

**Published:** 2025-07-14

**Authors:** Yanting Zhu, Yixiao Fu, Jiajia Han, Ruoming Wang, Xingshun Ma, Xiaomei Hu, Tao Li, Zhiwei Ma

**Affiliations:** 1 School of Biomedical Engineering, ShanghaiTech University, Shanghai, 201210 China; 2 Department of Psychiatry, The First Affiliated Hospital of Chongqing Medical University, Chongqing, 400016 China; 3 Key Laboratory of Major Brain Disease and Aging Research (Ministry of Education), Chongqing Medical University, Chongqing, 400016 China; 4 Precision Research Center for Refractory Diseases, Shanghai General Hospital, Shanghai Jiao Tong University School of Medicine, Shanghai, 201620 China; 5 Department of Neurology, The First Hospital of Yulin, Yulin, Shaanxi 719000 China; 6 Department of Abdominal Oncology, The Affiliated Hospital of Zunyi Medical University, Zunyi, Guizhou 563003 China; 7 Affiliated Mental Health Center and Hangzhou Seventh People’s Hospital, Zhejiang University School of Medicine, Hangzhou, Zhejiang 310013 China; 8 State Key Laboratory of Advanced Medical Materials and Devices, ShanghaiTech University, Shanghai, 201210 China; 9 Shanghai Clinical Research and Trial Center, Shanghai, 201210 China

**Keywords:** Neuroscience, Human behaviour

## Abstract

Adolescence is a pivotal phase of rapid brain development shaped by genetic and environmental factors, offering a critical window for identifying early indicators of psychiatric disorders. The Chongqing Adolescent Twin Study (CATS) explores genetic and environmental influences in 136 typically developing twins aged 12 to 19. This dataset includes multimodal MRI scans (structural, resting-state functional, and diffusion MRI) alongside extensive questionnaires on cognitive abilities, emotional and social behaviors, familial and parenting dynamics, sleep wellness, stress, anxiety, and depression. We describe the dataset in detail and systematically assess its quality. When benchmarked against the Lifespan Human Connectome Project Development (HCP-D) dataset, CATS meets or exceeds HCP-D standards in signal quality, tissue contrast, image sharpness, and head motion control. Preprocessing and imaging phenotype extraction facilitate broad reuse, and high phenotype correlations with HCP-D confirm reliability. This high-quality, multimodal resource provides a unique opportunity to investigate how genetic and environmental factors, along with age-related changes, shape adolescent brain structure, connectivity, and behavior, offering critical insights for precision medicine and early interventions in psychiatry.

## Background & Summary

Imaging genetics integrates neuroimaging and genetic data to investigate how genetic variation influences brain structure, connectivity, and behavior. This approach has demonstrated its utility in enhancing the early diagnosis and prognosis of psychiatric disorders, including attention-deficit/hyperactivity disorder^1^, bipolar disorder^2^, autism spectrum disorder^3^, depression^4^, and schizophrenia^5^. While it is challenging to directly observe the genetic origins of many psychiatric disorders from phenotypes^6^, neuroimaging-derived measures can serve as quantifiable intermediates between underlying genetic changes and external manifestations of diseases^7^, known as endophenotypes.

In this context, twin studies are particularly useful in imaging genetics for investigating the genetic and environmental influences on these endophenotypes. Monozygotic (MZ) twins share nearly 100% of their genetic similarity, while dizygotic (DZ) twins share on average 50%, with both types of twins sharing their early environment if rearing together. This natural experimental design enables researchers to disentangle the effects of genes and environment on brain structure and connectivity, providing critical insights into the heritability of endophenotypes^8^.

Studying imaging genetics in the adolescent brain is crucial because this period represents a unique convergence of rapid brain development and the initial onset of many psychiatric disorders. During adolescence, the brain undergoes significant structural and functional changes, particularly in the regions responsible for emotion regulation, decision-making, and social interactions^9^. These neurodevelopmental processes provide a critical window to observe how genetic variations influence brain maturation and how these interactions contribute to the emergence of psychiatric conditions^10^.

Adolescence spans a formative period during which environmental factors—such as stress, peer relationships, and family dynamics—profoundly influence mental health, often interacting with genetic predispositions in complex ways^11^. Investigating the genetic and neurobiological mechanisms underlying these interactions could help identify biomarkers for predicting the progression of psychiatric disorders. This, in turn, could enable earlier, more targeted interventions^12^, ultimately leading to precision medicine strategies tailored to an individual’s genetic and neurodevelopmental profile^13,14^. Moreover, examining these interactions at different stages of adolescence may reveal whether heritable and environmental factors shape brain structure, connectivity, and behavior differently as adolescents mature^15^, offering valuable insights into typical developmental trajectories and the identification of early risk factors.

The Chongqing Adolescent Twin Study (CATS) was designed and implemented to examine these genetic and environmental effects on brain structure, connectivity, and behavior during this critical developmental stage^16–18^. This dataset includes both brain imaging and non-imaging data collected from typically developing MZ and DZ adolescent twins. The brain imaging data includes structural MRI (sMRI), resting-state functional MRI (rsfMRI), and diffusion MRI (dMRI), while the non-imaging data contains over 600 items across a wide range of questionnaires, providing rich information on cognitive abilities, emotional and social behaviors, familial and parenting dynamics, sleep wellness, stress, anxiety, and depression.

Initial analyses of this comprehensive dataset have yielded significant advancements in revealing the heritability of cortical surface area, resting-state functional connectivity, and behavior during adolescence. Specifically, high heritability was found for total cortical surface area^18^. Furthermore, studies confirmed that the phenotypic correlation between regional cortical surface area and verbal intelligence was largely due to genetic effects rather than unshared environmental influences or measurement error^19^. Regarding resting-state functional connectivity, analyses revealed that heritability varies across different resting-state networks, with stronger genetic control in sensory networks and relatively weaker control in cognitive networks^17^. In terms of adolescent behavior, emotional problems were largely influenced by genetics, while peer problems were predominantly shaped by environmental factors^16^.

While the CATS dataset has already provided valuable insights into genetic and environmental influences on adolescent brains and behavior, it remains a rich resource with significant untapped potential for future research. Priority areas include examining the genetic underpinnings of complex structural metrics^20^, the dynamic nature of functional connectivity^21^, and the interplay between brain development and cognitive, emotional, and social behaviors. Another important direction involves investigating how these genetic and environmental factors evolve across adolescence, which can be pursued by analyzing the CATS dataset on its own or in combination with other adolescent or adult datasets^20–22^.

In the present work, we provide a comprehensive description of the CATS dataset. We assessed the quality of the data and conducted a comparison with a subset of age-matched data from the Lifespan Human Connectome Project Development (HCP-D)^20^. To facilitate future reuse of the CATS dataset, we have performed preprocessing and extracted imaging phenotypes across multiple modalities, using two widely recognized pipelines for each modality. This dataset, along with its derivatives, offers an invaluable resource for investigating the interplay of genetic and environmental factors on various dimensions of brain structure, connectivity, and behavior in Chinese adolescents.

## Methods

### Participants

**Fig. 1:**
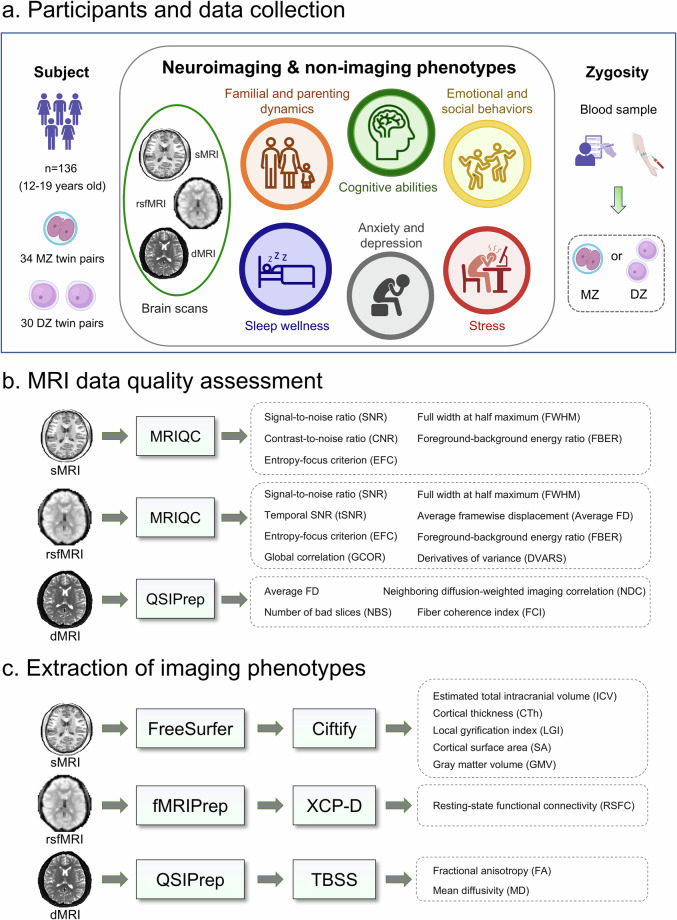
Overview of the Chongqing Adolescent Twin Study (CATS). **(a)** Participants and data collection. We enrolled 136 adolescent twins; zygosity was confirmed for 128 individuals, comprising 34 monozygotic (MZ) and 30 dizygotic (DZ) pairs. The data collection includes structural MRI (sMRI), resting-state functional MRI (rsfMRI), diffusion MRI (dMRI), and questionnaires covering cognition, emotional and social behaviors, family environment, sleep, stress, anxiety, and depression. **(b)** MRI data quality assessment: Image quality was evaluated with MRIQC for sMRI and rsfMRI data, and with QSIPrep for dMRI. Key quality metrics for each modality are displayed. **(c)** Imaging phenotype extraction: sMRI data were processed with FreeSurfer and Ciftify; rsfMRI data with fMRIPrep and XCP-D; and dMRI data with QSIPrep followed by Tract-Based Spatial Statistics (TBSS). The resulting phenotypic measures for each modality are listed.

A total of 136 typical developing twins aged 12–19 years (mean ± SD = 15.71 ± 1.61 years, 54% female) from local schools in Chongqing, China, participated in this study (see Fig. 1 for a schematic overview). Written informed consent, including permission for data sharing, was obtained from all participants and their parents. Ethics approval for the CATS was granted by Chongqing Medical University, and the reanalysis of the CATS dataset was approved by the Research Ethics Review Committee of ShanghaiTech University. All participants were free from psychiatric disorders, nervous system diseases, and severe physical diseases. All twin pairs were reared together except for one pair. Each subject completed questionnaires for non-imaging phenotyping, assessing cognitive abilities, emotional and social behaviors, familial and parenting dynamics, sleep wellness, stress, anxiety, and depression. Among the 136 participants, 128 had zygosity test results based on short tandem repeats and amelogenin^23^, comprising 34 pairs of MZ twins and 30 pairs of DZ twins. Of these, 120 participants (33 MZ pairs and 27 DZ pairs) had complete data of sMRI, rsfMRI, and dMRI. Detailed demographic information is provided in Table 1.

**Table 1:** Summary of the demographic information of the Chongqing Adolescent Twin Study (CATS) dataset.

Type	Sample size	Sex		Age					Number of subjects with zygosity results		
		M	F	12-13	14-15	16-17	18-19	mean ±SD	Total	MZ	DZ
Non-imaging	136	63	73	14	32	74	16	15.71 ± 1.61	128	68	60
sMRI	129	59	70	10	30	73	16	15.83 ± 1.51	122	66	56
rsfMRI	128	58	70	10	30	72	16	15.83 ± 1.51	121	66	55
dMRI	129	59	70	10	30	73	16	15.83 ± 1.51	122	66	56

**Abbreviations:** sMRI, structural MRI; rsfMRI, resting-state functional MRI; dMRI, diffusion MRI; MZ, monozygotic; DZ, dizygotic.

### MRI acquisition

For the CATS subjects, their brain MRI data were collected using a 3 T scanner (Signa, GE Medical Systems, Waukesha, WI). All the participants were asked to lie still with their eyes closed. The T1w sMRI data were acquired using the 3DT1 sequence with the following parameters: repetition time (TR) = 6.1 ms; echo time (TE) = 2.8 ms; flip angle = 12°; matrix size = 256 × 256; field of view (FOV) = 24 cm × 24 cm; slice number = 166; slice thickness = 1.2 mm. The rsfMRI data were acquired using gradient-echo echo-planar imaging (EPI) sequence with the following parameters: TR = 3000 ms; TE = 30 ms; flip angle = 90°; matrix size = 64 × 64; FOV = 24 cm × 24 cm; slice number = 33; slice thickness = 3.5 mm. 240 volumes were acquired for each rsfMRI run. Subjects were instructed to respond post-rsfMRI as a measure to confirm wakefulness and prevent them from falling asleep throughout this experiment. The dMRI data were acquired using spin-echo EPI sequence with the following parameters: TR = 13.8 s; TE = 86.7 ms; flip angle = 90°; matrix size = 256 × 256; FOV = 24 cm × 24 cm; slice number = 37; slice thickness = 3 mm. Each run consisted of 26 diffusion-weighted volumes, which included one unweighted reference volume (b = 0 s/mm^2^) and 25 diffusion-weighted volumes acquired at b = 1000 s/mm^2^. These 25 gradient vectors were unit-length, non-colinear, and uniformly distributed on the sphere, providing angular coverage suitable for reliable tensor estimation.

### Non-imaging phenotyping

Table 2 catalogs various questionnaires employed to assess CATS participants, encompassing cognitive abilities, emotional and social behaviors, familial and parenting dynamics, sleep wellness, stress, as well as anxiety and depression. The table also distinguishes between the versions of questionnaires, specifying whether they are self-reported by the adolescents, completed by their parents, or jointly by both parties. The details of these questionnaires are as follows:

1. Cognitive abilities

- The *Chinese revision of the Wechsler Intelligence Scale for Children (C-WISC)*^24^ is an assessment of cognitive functioning and intelligence, including verbal comprehension, working memory, processing speed, attention, and reasoning skills.
- The *Wisconsin Card Sorting Test (WCST)*^25^ is an assessment of executive functions of the frontal lobe, particularly cognitive flexibility and working memory.

(2) Emotional and social behaviors

- The *Strengths and Difficulties Questionnaire (SDQ)*^26^ evaluates emotional and behavioral issues for children aged 11 and above.
- The *Everyday Feelings Questionnaire (EFQ)*^27^ assesses the subject’s feelings, and a higher score represents a better feeling.
- The *Index of General Affect (IGA)*^28^ assesses an individual’s sense of happiness and life satisfaction, with higher scores indicating a stronger sense of happiness experienced by the individual.
- The *Child Behavior Checklist (CBCL)*^29^ is a comprehensive scale to assess behaviors and emotional issues in adolescents, identifying potential problems such as anxiety, depression, aggression, and attention issues.
- The *Risk Behavior Questionnaire-Adolescent (RBQ-A)*^30^ captures both the incidence and frequency of risky behaviors over the preceding month. Consequently, a higher total score on the RBQ-A correlates with a greater level of risk-taking behavior among adolescents.
- The *Children’s version of the Eysenck Personality Questionnaire (EPQ)*^31^ is for assessing the personality characteristics of adolescents.

(3) Familial and parenting dynamics

- The *Parenting Styles and Dimensions Questionnaire (PSDQ)*^32^ assesses various parenting styles, including authoritative, permissive, and authoritarian. A higher score signifies a stronger alignment with the corresponding parenting style.
- The *McMaster Family Assessment Device - General Functioning Scale (FAD-GFS)*^33^ evaluates key aspects of family dynamics, such as the coordination of activities, resilience during crises, and levels of mutual trust. Higher scores on this scale typically indicate poorer family functioning.
- The *Family Life Questionnaire (FLQ)*^27^ is crafted to evaluate the behavioral dynamics among family members as well as the approaches to encouragement and discipline within the family. A higher score on the questionnaire reflects a more harmonious family environment.
- The *Family Adaptability and Cohesion Evaluation Scale, 2nd edition, Chinese version (FACESII-CV)*^34^ measures the adaptability and cohesion aspects of family functioning. It is designed to accurately and effectively evaluate these dimensions in Chinese families, with higher scores representing greater adaptability and cohesion.

(4) Sleep wellness

- The *Pittsburgh Sleep Quality Index (PSQI)*^35^ is a self-assessment scale that enables individuals to rate their sleep quality, encompassing various aspects such as sleep duration, sleep disturbances, and daytime dysfunction.

(5) Stress

- The *General Health Questionnaire-12 (GHQ-12)*^36^ evaluates the psychological well-being of the parents. This widely-used screening tool is designed to identify short-term changes in mental health, specifically focusing on the ability to carry out daily activities and the presence of distressing psychological symptoms.
- The *Family Stress Questionnaire (FSQ)*^27^ is an assessment designed to capture a spectrum of stressors experienced by family members, including occupational pressures, economic status, psychological and physiological well-being, as well as the strain arising from interactions with the external environment. Higher scores on the FSQ indicate elevated levels of stress.
- The *Simplified Coping Style Questionnaire (SCSQ)*^37^ measures whether individuals adopt positive or negative coping styles when facing stress.

(6) Anxiety and depression

- The *Self-Rating Anxiety Scale (SAS)*^38^ measures anxiety levels through self-report, helping individuals reflect on their feelings and symptoms.
- The *Beck Depression Inventory (BDI)*^39^ is a self-assessment tool aimed at identifying the presence and severity of depressive symptoms in individuals.

**Table 2:** Chongqing Adolescent Twin Study (CATS) non-imaging phenotypes.

Domain	Measurement Source	Version
**Cognitive abilities**		
	Chinese revision of Wechsler Intelligence Scale for Children (C-WISC)^24^	Adolescent
	Wisconsin Card Sorting Test (WCST)^25^	Adolescent
**Emotional and social behaviors**		
	Strengths and Difficulties Questionnaire (SDQ)^26^	Adolescent, father or mother
	Everyday Feelings Questionnaire (EFQ)^27^	Father or mother
	Index of General Affect (IGA)^28^	Father or mother
	Child Behavior Checklist (CBCL)^29^	Adolescent, father or mother
	Risk Behavior Questionnaire-Adolescent (RBQ-A)^30^	Adolescent
	Eysenck Personality Questionnaire (EPQ)^31^	Adolescent
**Familial and parenting dynamics**		
	Parenting Style and Dimension Questionnaire (PSDQ)^32^	Father and mother
	McMaster Family Assessment Device - General Functioning Scale (FAD-GFS)^33^	Father or mother
	Family Life Questionnaire (FLQ)^27^	Father or mother
	Family Adaptability and Cohesion Evaluation Scale 2nd edition Chinese Version (FACESII-CV)^34^	Father or mother
**Sleep wellness**		
	Pittsburgh sleep quality index (PSQI)^35^	Adolescent
**Stress**		
	General Health Questionaire-12 (GHQ-12)^36^	Father and mother
	Family Stress Questionnaire (FSQ)^27^	Father or mother
	Simplified Coping Style Questionnaire (SCSQ)^37^	Adolescent
**Anxiety and depression**		
	Self-Rating Anxiety Scale (SAS)^38^	Adolescent
	Beck Depression Inventory (BDI)^39^	Adolescent

### MRI data quality assessment

The quality of the CATS dataset was assessed by comparing 17 data quality metrics to those from the Lifespan Human Connectome Project Development (HCP-D)^20^, a well-established adolescent neuroimaging dataset. To ensure an age-matched comparison, 336 subjects aged 12 to 19 years (mean ± SD = 15.13 ± 1.93 years, 52% female) were selected from the HCP-D dataset, and their image quality metrics were derived accordingly. Statistical analyses were conducted using OriginPro (v2024; https://www.originlab.com), with Welch’s t-test applied for metrics following a normal distribution with unequal variance, and the Mann-Whitney U test for metrics with non-normal distributions. The image quality metrics are described as follows.

For the sMRI data, five quality metrics computed by the MRIQC (v0.16.1)^40^ were used for quality assessment:

1. *Signal-to-noise ratio (SNR)*^41^. SNR measures the level of the brain signal relative to background noise (e.g., air in the image). Higher SNR indicates that the brain signal is much stronger than noise, reflecting better image quality.
2. *Full width at half maximum (FWHM)* of image smoothness^42^. MRIQC computes FWHM by calling AFNI’s 3dFWHMx^43^, which fits a Gaussian model to the volume’s spatial autocorrelation. Lower FWHM indicates a sharper image with more distinct anatomical boundaries.
3. *Contrast-to-noise ratio (CNR)*^44^. CNR is used to evaluate the separation between the tissue distributions of gray matter and white matter. A higher CNR indicates a clearer distinction between tissues, implying better image quality.
4. *Foreground-background energy ratio (FBER)*^45^. FBER is the ratio of the mean energy within the head to the mean energy in the surrounding air mask. A higher FBER means the signal within the head is relatively stronger than the background air, suggesting better image quality.
5. *Entropy-focus criterion (EFC)*^46^. EFC utilizes the Shannon entropy of voxel intensities to detect ghosting and blurring resulting from head motion. Lower EFC indicates fewer artifacts such as ghosting or blurring, hence better image quality.

For the rsfMRI data, eight quality metrics computed by the MRIQC (v0.16.1)^40^ were used for quality assessment:

1. *SNR*. This metric was described in the sMRI data quality assessment paragraph above.
2. *Temporal SNR (tSNR)*^47^. The tSNR is calculated by first generating a voxelwise tSNR map, which is obtained by dividing the mean BOLD signal across time by its corresponding temporal standard deviation map. Then, the median value of this tSNR map is determined to establish the tSNR. A higher tSNR indicates that the signal is more consistent over time relative to fluctuations, implying better data quality.
3. *FWHM* of image smoothness. This metric was described in the sMRI data quality assessment paragraph above.
4. *Average framewise displacement (Average FD)*^48^. FD is calculated by summing the absolute magnitudes of translational and rotational head movements between each consecutive fMRI frame. Rotational movements are transformed into their translational equivalents across the curvature of a standard sphere, which is assumed to be 50 mm in radius, roughly approximating the human brain size. For each subject, an average FD value is computed and reported to provide a measure of head motion during the rsfMRI session. A lower average FD indicates less head motion, which typically corresponds to better data quality.
5. *Derivatives of variance (DVARS)*^49^. DVARS quantifies the rate of change in BOLD signal intensity across the whole brain between successive time points. It is used to assess data quality by highlighting sudden signal intensity changes, aiding in the detection of motion-related and physiological noise in the rsfMRI data. For each subject, the mean DVARS across time was reported. Lower DVARS generally suggests fewer abrupt signal changes.
6. *Global correlation (GCOR)*^50^. GCOR is the average correlation between all pairs of voxel time series within the brain. It highlights differences in the data caused by motion, physiological noise, or imaging artifacts. A smaller GCOR indicates that the data are less dominated by global artifacts, implying better quality.
7. *FBER*. This metric was described in the sMRI data quality assessment paragraph above.
8. *EFC*. This metric was described in the sMRI data quality assessment paragraph above.

For the dMRI data, four quality metrics computed by the QSIPrep (v0.17.0)^51^ were used for quality assessment:

1. *Neighboring diffusion-weighted imaging correlation (NDC)*. NDC measures the spatial correlations between dMRI volumes that are acquired from nearby sampling points in q-space. High NDC values indicate that neighboring volumes are consistent with each other, reflecting better data quality.
2. *Average FD*. This metric was described in the rsfMRI data quality assessment paragraph above.
3. *Number of bad slices (NBS)*^52^. NBS is detected by identifying signal dropouts and comparing each slice with its neighboring slices. A smaller NBS indicates fewer corrupted slices, and thus better data quality.
4. *Fiber coherence index (FCI)*^53^. FCI quantifies the alignment of neighboring fiber orientations, weighted by anisotropy values, and is used to ensure correct b-vector orientation for accurate fiber tractography. A higher FCI indicates that the estimated fibers are more coherently aligned, suggesting better dMRI quality.

### Extraction of imaging phenotypes

To facilitate further reuse of the CATS dataset, we extracted five sMRI-derived imaging phenotypes, one rsfMRI-derived imaging phenotype, and two dMRI-derived imaging phenotypes. Before extraction, data quality outliers for each modality were first excluded. Since rsfMRI and dMRI preprocessing depend on usable sMRI images, subjects with poor sMRI quality were excluded from further analyses of sMRI, rsfMRI, and dMRI. The sMRI quality was evaluated using FreeSurfer’s Euler number (FreeSurfer v6)^54^, which summarizes cortical surface reconstruction quality and reflects the quality of the sMRI image^55^. Subjects with Euler numbers exceeding the third quartile (Q3) plus three times the interquartile range (IQR) were excluded as extreme outliers^56,57^. For rsfMRI, subjects with an average FD greater than 0.2 mm were excluded^49^. For dMRI, subjects who were extreme outliers in terms of NDC or average FD were excluded.

The raw sMRI data were processed using FreeSurfer. The individual-level sMRI-derived imaging phenotypes extracted from FreeSurfer outputs included *estimated total intracranial volume (ICV)*, *cortical thickness (CTh)*, *cortical surface area (SA)*, *gray matter volume (GMV)*, and *local gyrification index (LGI)*. ICV encompasses all intracranial volumes of gray matter, white matter, and cerebrospinal fluid. CTh measures the distance between gray-white matter and pial boundaries in specific cortical regions. SA measures the area of the outer (pial) surface of specific cortical regions. GMV quantifies the volume of gray matter within specific brain regions. LGI quantifies cortical folding at each cortical location by measuring the ratio of the buried (sulcal) SA to the outer (pial) SA. These metrics provide insights into cortical structure, allowing investigations into developmental and pathological changes in brain morphology. The Destrieux atlas was used to obtain regional values for CTh, SA, GMV, and LGI^58^. To enable comparison with the reference dataset (the age-matched subgroup of 336 subjects from the HCP-D dataset), we transformed FreeSurfer outputs to standard CIFTI grayordinate space using Ciftify (v2.3.3)^59^. The HCP-D sMRI data were preprocessed using the HCP structural pipeline (v4.3.0)^60^. We calculated the mean and variance of CTh for both datasets and assessed their relationship using Pearson correlation.

The raw rsfMRI data were preprocessed using fMRIPrep (v22.1.1)^61^ and XCP-D (v0.4)^62^. The fMRIPrep steps here included brain extraction, head motion correction, slice timing correction, susceptibility distortion correction, and brain normalization. The XCP-D steps included nuisance signal regression (six head motion parameters, cerebrospinal fluid signal, white matter signal, and global signal) and bandpass filtering (0.01–0.08 Hz). The HCP-D rsfMRI data were preprocessed using the HCP functional pipeline (v4.3.0)^60^. Preprocessed rsfMRI time series from the XCP-D or HCP outputs in the standard CIFTI grayordinate space were used to calculate individual-level *resting-state functional connectivity (RSFC)* matrices based on Gordon’s parcellation of 333 regions^63^. RSFC measures the statistical relationships (e.g., correlations) between different brain regions over time, providing insight into functional communication across the brain. We then calculated the mean and variance of RSFC for each dataset and computed the Pearson correlation of mean RSFC between the two datasets.

The raw dMRI data were reoriented to standard orientation and preprocessed using the QSIPrep pipeline (v0.17.0)^51^. Scilpy (v1.1.0) was used to extract the b = 1000 s/mm^2^ shell, and diffusion tensor model fitting was conducted using FSL (v6.0)^64^. The resulting *fractional anisotropy (FA)* and *mean diffusivity (MD)* images were analyzed using Tract-Based Spatial Statistics (TBSS)^65^, and mean values were extracted within the white matter regions defined by the Johns Hopkins University atlas^66^. The MD values were multiplied by 10,000 to convert to units of 10^−4^ mm^2^/s. FA reflects the degree to which diffusion is directionally constrained, while MD measures the overall magnitude of diffusion. Both are commonly used to examine the microstructural properties of white matter. For the HCP-D dMRI data, similar preprocessing steps were used, but the b = 1500 s/mm^2^ shell was utilized instead. We calculated the mean and variance of FA and MD for each dataset and computed the Pearson correlation of mean FA and MD between the two datasets.

To provide users with alternative preprocessing options, we employed additional processing pipelines for each imaging modality. For the raw sMRI data, we used the Computational Anatomy Toolbox (v12)^67^ to extract CTh and GMV. The rsfMRI data were alternatively processed using DPABI (v8.2)^68^, which performed slice timing correction, motion correction, sMRI-rsfMRI co-registration, brain segmentation, nuisance signal regression (the parameters of Friston 24-parameter head motion model^69^, cerebrospinal fluid signal, white matter signal, and global signal), brain normalization, spatial smoothing, bandpass filtering, and RSFC calculation using the same parcellation scheme as our primary processing methods. For the raw dMRI data, we implemented the FSL diffusion tensor imaging pipeline, which included reorientation to standard orientation, eddy current correction^70^, motion correction, diffusion tensor model fitting (using the b = 1000 s/mm^2^ shell), and TBSS analysis^70^. Regional FA and MD values were calculated using the Johns Hopkins University atlas^66^, consistent with our primary processing approach.

### Data anonymization

To protect participant privacy, we thoroughly de-identified the CATS dataset. First, we manually checked all data to ensure the absence of personally identifiable information (e.g., name, date of birth, or date of scan). For imaging data, we performed facial anonymization using FSL’s fsl_deface and then visually inspected the resulting images^71^. Any images that were not successfully defaced were processed with pydeface instead^72^, ensuring that facial features could not be reconstructed while preserving critical brain structures for analysis. We also carefully reviewed metadata (including NIfTI file headers and JSON files) to confirm that no personally identifiable information remained in either raw or derived imaging data. Finally, we conducted thorough manual checks of non-imaging phenotyping data to remove any direct identifiers or potentially identifying details, thereby maintaining strict confidentiality throughout the dataset.

## Data Records

### Dataset access and organization

The CATS dataset is accessible through the Brain Science Data Center website of the Chinese Academy of Sciences (10.12412/BSDC.1736128526.40001)^73^. Instructions for requesting access are provided in the *Usage Notes* section of this manuscript. Due to the dataset’s substantial size, users are advised to utilize download tools optimized for handling large files.

Fig. 2 presents the top-level folder hierarchy of the dataset. It includes raw (unprocessed) imaging data, derived imaging data, and non-imaging phenotyping data. The detailed structure of each subfolder within the derived imaging data directory is illustrated in Figs. S1–S8.

### Imaging data

**Fig. 2:**
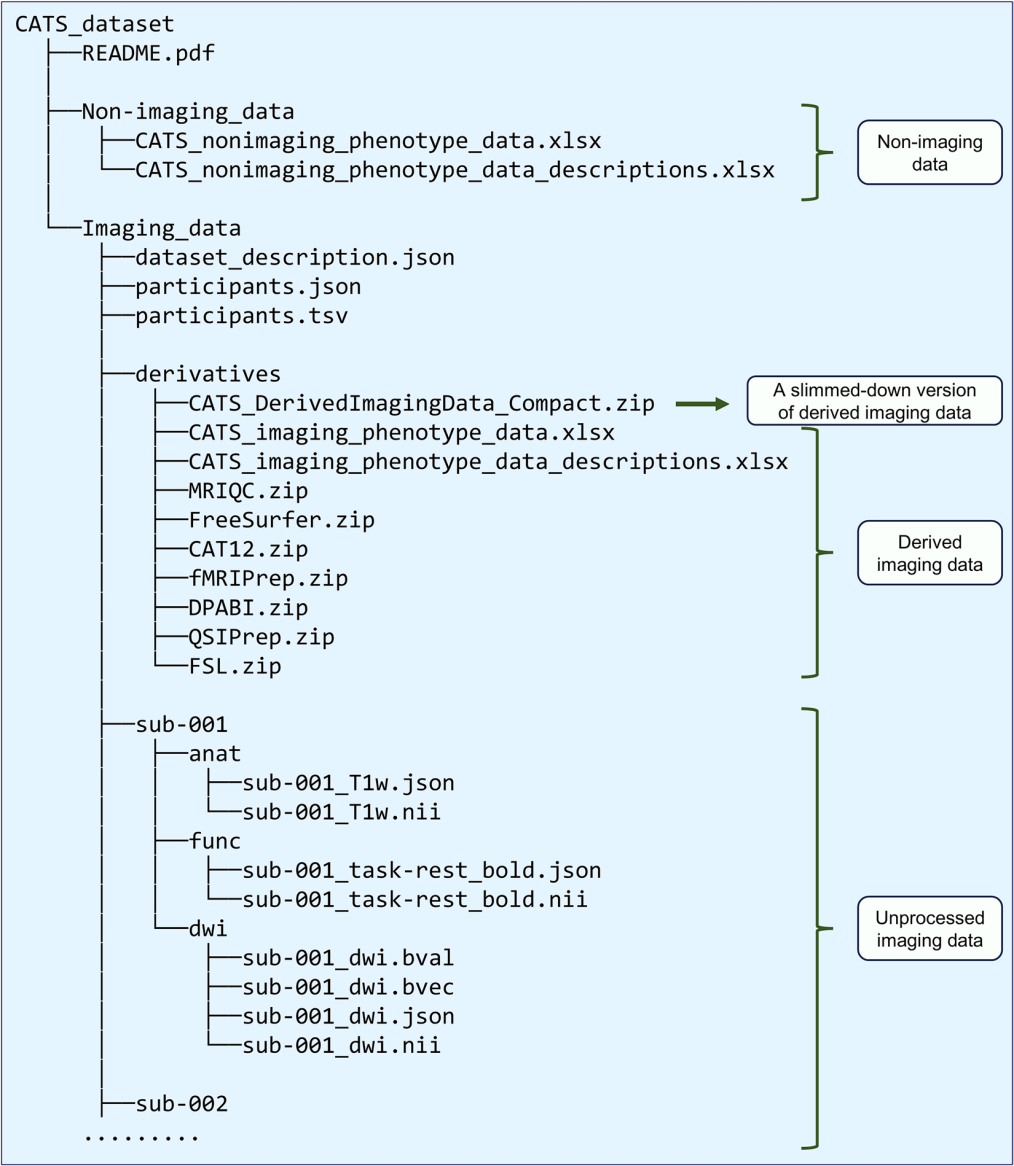
Folder structure of the Chongqing Adolescent Twin Study (CATS) dataset. Raw imaging data—together with quality control outputs and preprocessed images—are organized according to the Brain Imaging Data Structure (BIDS) specification. The file *CATS_DerivedImagingData_Compact.zip* is a lightweight compressed archive that contains only the key subject-level imaging phenotype files, allowing rapid download and inspection of derived metrics. Extracted imaging phenotype values are stored in *CATS_imaging_phenotype_data.xlsx*, while non-imaging measures are provided in *CATS_nonimaging_phenotype_data.xlsx*.

The imaging data adhere to the Brain Imaging Data Structure (BIDS) specification^74^. Basic demographic information such as age, sex, handedness, and family ID of the participants can be found in the file *participants.tsv*, with column descriptions provided in participants.json. Participant data are organized in subfolders named after their corresponding subject IDs. Within each subject folder, raw data from different imaging modalities are stored in the *anat* (sMRI), *func* (rsfMRI), and *dwi* (dMRI) subfolders, respectively (Fig. 2). Each scan within these modality folders is stored as a NIfTI file (*.nii*), accompanied by a JSON file (*.json*) that provides MRI acquisition parameters. For the sMRI data, facial features have been obscured to protect participant privacy^75^. For the dMRI data, gradient orientation information is provided in files named *_dwi.bvec and *_dwi.bval. All available data are included in the dataset, regardless of the quality assessment results.

The image quality control results and the preprocessed outputs from the primary and alternative processing pipelines are provided in subfolders under the derivatives directory. The compressed archive *CATS_DerivedImagingData_Compact.zip*, a slimmed-down bundle of derived imaging data, contains only the essential subject-level imaging phenotype files (Fig. S1), making it a lightweight download for rapid access to derived imaging metrics. The compressed archive *MRIQC.zip* contains image quality control results for the sMRI and rsfMRI data using MRIQC (Fig. S2). Within the compressed archive *Freesurfer.zip*, the T1w subfolder contains FreeSurfer outputs of the sMRI data in their native space, and the CIFTI subfolder includes FreeSurfer outputs resampled to the standard CIFTI grayordinate space (Fig. S3). The compressed archive *CAT12.zip* contains outputs from the Computational Anatomy Toolbox for sMRI data (Fig. S4). The compressed archive *fMRIPrep.zip* stores the fMRIPrep-preprocessed results of the rsfMRI data (Fig. S5), while the compressed archive *DPABI.zip* contains the DPABI-preprocessed results of the rsfMRI data (Fig. S6). The compressed archive QSIPrep.zip includes dMRI image quality control results and primary processing pipeline outputs generated using QSIPrep, Scilpy, and TBSS (Fig. S7). The compressed archive *FSL.zip* stores dMRI alternative processing pipeline outputs generated using FSL modules (Fig. S8). Extracted imaging phenotype values are also stored in the file *CATS_imaging_phenotype_data.xlsx*, with variables and properties described in *CATS_imaging_phenotype_descriptions.xlsx*.

### Non-imaging phenotyping data

The non-imaging phenotyping data include zygosity information and questionnaire results related to cognitive abilities, emotional and social behaviors, familial and parenting dynamics, sleep wellness, stress, as well as anxiety and depression. These data are provided in the file *CATS_nonimaging_phenotype_data.xlsx*, with variables and properties described in *CATS_nonimaging_phenotype_descriptions.xlsx* (Fig. 2). All participant-identifiable information has been removed to ensure privacy.

## Technical Validation

### Imaging data

**Fig. 3:**
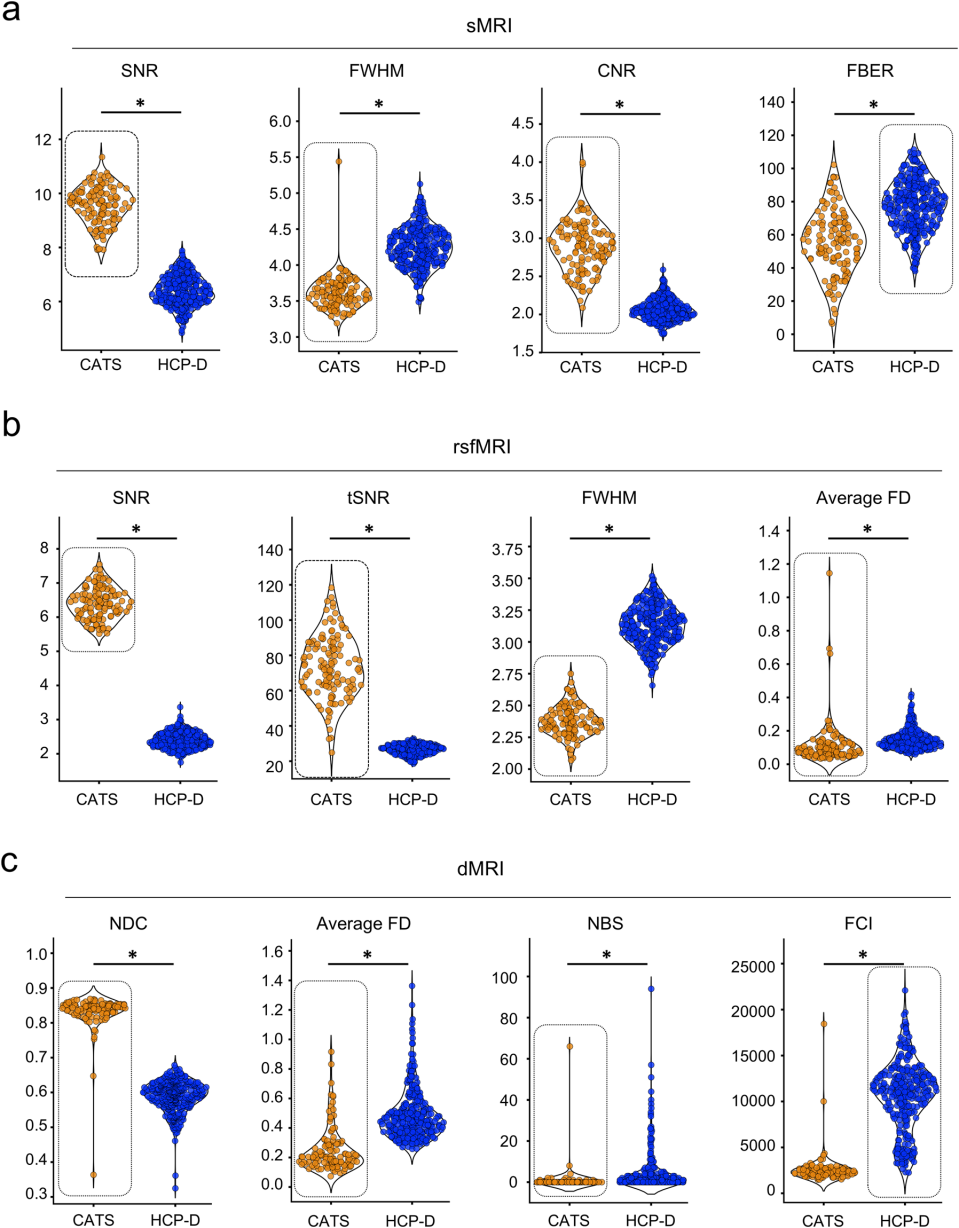
Data quality comparison between the Chongqing Adolescent Twin Study (CATS) and Lifespan Human Connectome Project Development (HCP-D) datasets. This figure compares the data quality metrics of the CATS with those of the HCP-D datasets. For each quality control metric, the beeswarm plot circled with a dashed box indicates the dataset with superior quality. An asterisk (*) signifies that the means of the two distributions are significantly different (p < 0.05). **(a)** Structural MRI (sMRI) quality control metrics: Signal-to-noise ratio (SNR), full width at half maximum (FWHM), contrast-to-noise ratio (CNR), and foreground-background energy ratio (FBER). **(b)** Resting-state functional MRI (rsfMRI) quality control metrics: SNR, temporal signal-to-noise ratio (tSNR), FWHM, and average framewise displacement (Average FD). **(c)** Diffusion MRI (dMRI) quality control metrics: Neighboring diffusion-weighted imaging correlation (NDC), Average FD, number of bad slices (NBS), and fiber coherence index (FCI).

We validated the CATS imaging dataset quality by comparing it with the HCP-D dataset using established metrics. For sMRI, CATS demonstrated significantly higher SNR, CNR, and FWHM (Fig. 3a), indicating better image quality and sharper anatomical delineation. For rsfMRI, CATS showed significantly improved SNR, tSNR, FWHM, average FD, and EFC (Figs. 3b and S9b), reflecting enhanced image quality, sharpness, and head motion control. For dMRI, CATS exhibited significantly better NDC, average FD, and NBS than HCP-D (Fig. 3c), suggesting improved spatial consistency among neighboring diffusion-weighted volumes, reduced head motion artifacts, and fewer corrupted slices. Although HCP-D outperformed CATS in certain sMRI (FBER, EFC; Figs. 3a, S9a), rsfMRI (DVARS, GCOR, FBER; Fig. S9b), and dMRI metrics (FCI; Fig. 3c), the overall comparison confirms that CATS meets or exceeds HCP-D standards across all imaging modalities.

**Fig. 4:**
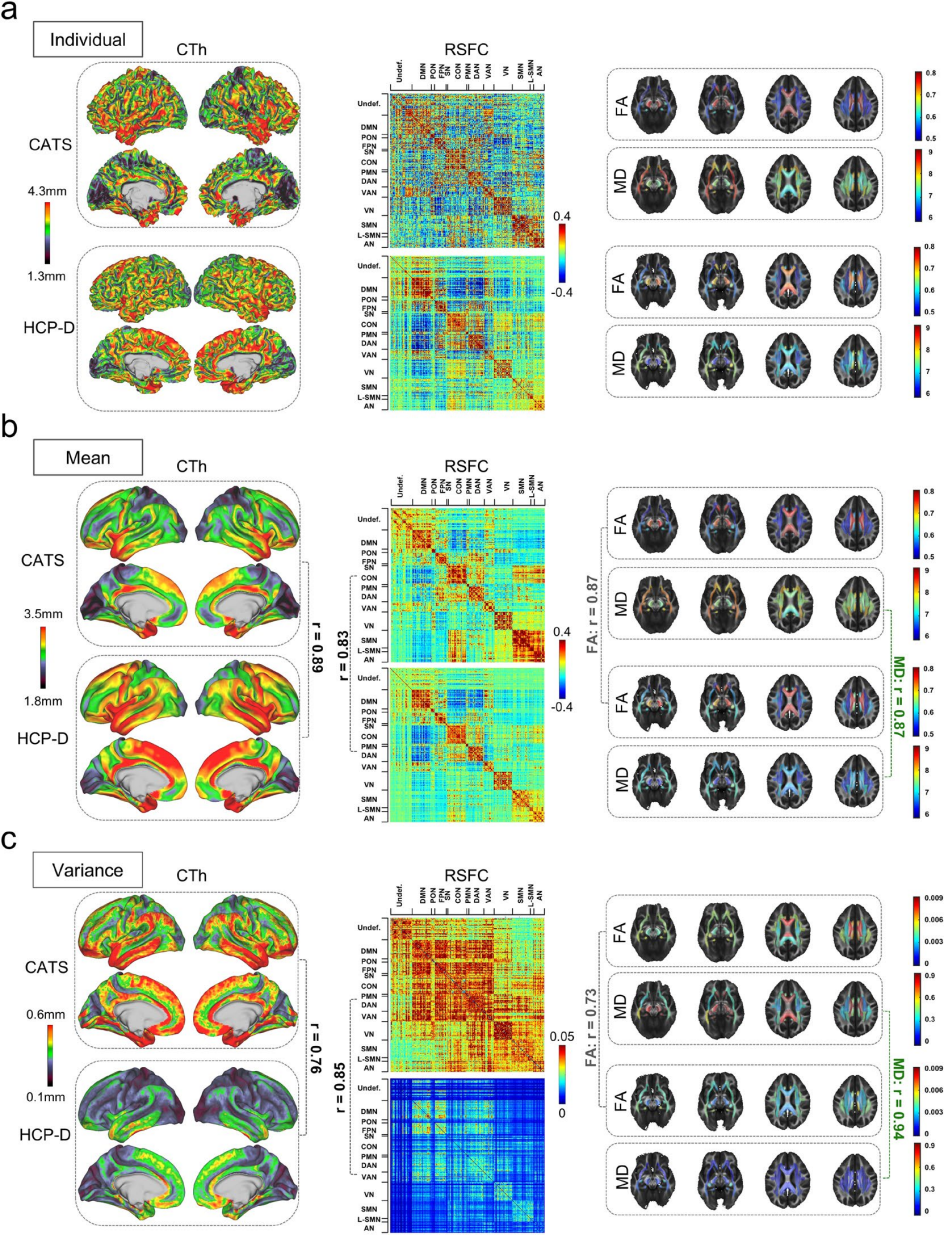
Comparison of extracted imaging phenotypes between the Chongqing Adolescent Twin Study (CATS) and the Lifespan Human Connectome Project Development (HCP-D) datasets. **(a)** Maps of individual-level cortical thickness (CTh; first column), resting-state functional connectivity (RSFC; second column), fractional anisotropy (FA; third column), and mean diffusivity (MD; third column) for a representative subject from the CATS dataset and a representative subject from the HCP-D dataset. **(b)** Group-average maps of CTh (first column), RSFC (second column), FA (third column), and MD (third column) for the CATS and HCP-D datasets. The spatial correlations between the two datasets for these group-average maps are: CTh, 0.89; RSFC, 0.83; FA, 0.87; MD, 0.87. **(c)** Variance maps of CTh (first column), RSFC (second column), FA (third column), and MD (third column) for the CATS and HCP-D datasets. The spatial correlations between the variance maps of the two datasets are: CTh, 0.76; RSFC, 0.85; FA, 0.73; MD, 0.94. In this figure, the unit of MD is 10^−4^ mm^2^/s. For visualization purposes, FA and MD maps display each white matter region of interest (ROI) in full size instead of only the Tract-Based Spatial Statistics (TBSS) skeleton. **Abbreviations**: Undef., undefined; DMN, default-mode network; PON, parieto-occipital network; FPN, frontoparietal network; SN, salience network; CON, cingulo-opercular network; PMN, parietal memory network; DAN, dorsal attention network; VAN, ventral attention network; VN, visual network; SMN, somatomotor network; L-SMN, lateral somatomotor network; AN, auditory network.

We also evaluated extracted imaging phenotypes from the three modalities. Comparing a representative CATS subject to a representative HCP-D subject revealed similar spatial patterns across sMRI, rsfMRI, and dMRI measures (Fig. 4a). At the group level, the mean and variance of CTh, RSFC, FA, and MD showed strong concordance with the HCP-D dataset. Specifically, the spatial correlation between CATS and HCP-D was 0.89 for mean CTh and 0.76 for CTh variance (Fig. 4bc), 0.83 for mean RSFC and 0.85 for RSFC variance (Fig. 4bc), 0.87 for mean FA and 0.73 for FA variance (Fig. 4bc), and 0.87 for mean MD and 0.94 for MD variance (Fig. 4bc). These high inter-dataset correlations demonstrate that the CATS dataset reliably captures well-established neuroimaging phenotypes.

**Fig. 5:**
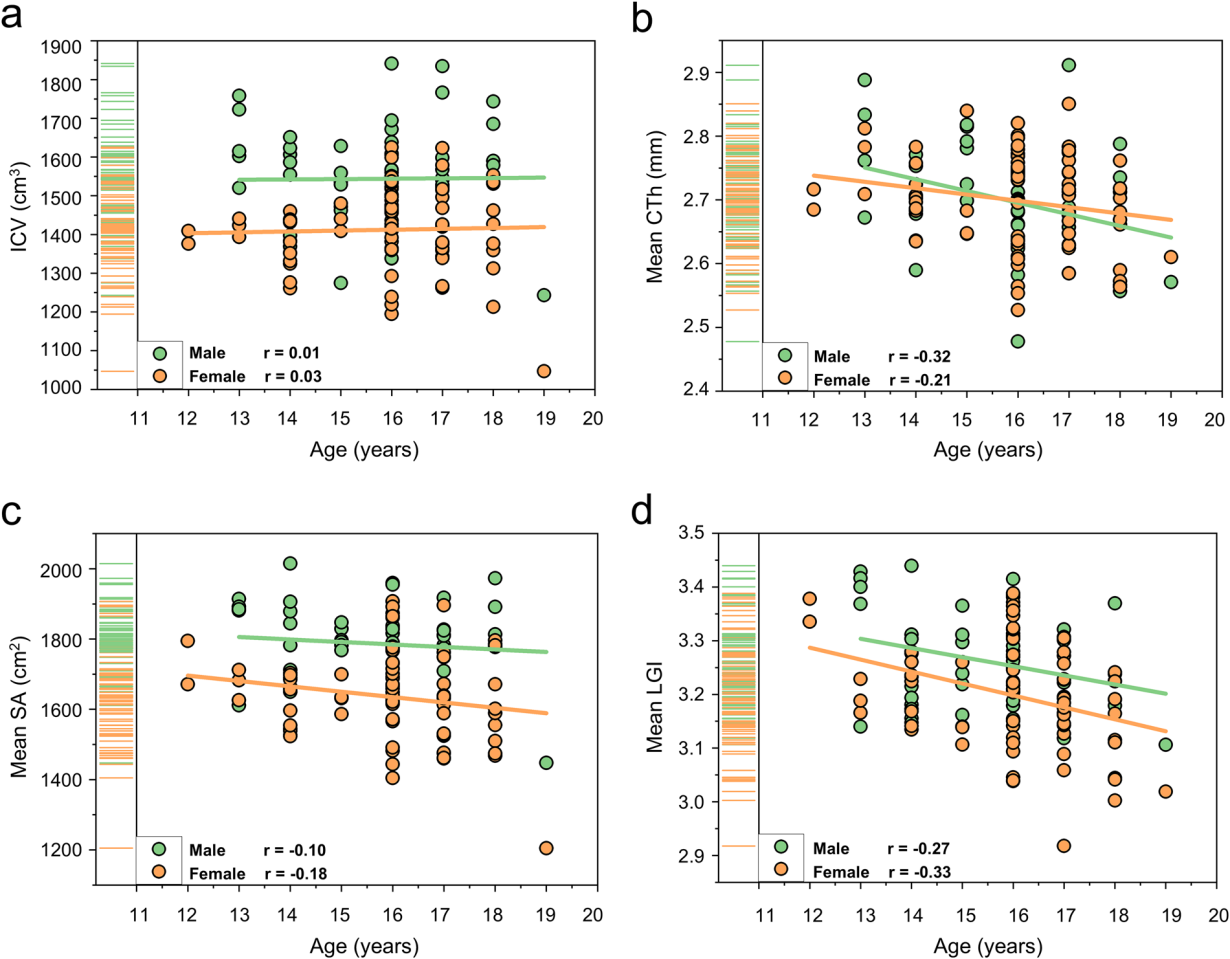
Relationship between structural MRI (sMRI) imaging phenotypes, age, and sex. Scatter plots illustrating the correlations between age (x-axis) and various sMRI imaging phenotypes (y-axis), with data points colored by sex. **(a)** Estimated total intracranial volume (ICV). **(b)** Mean cortical thickness (CTh). **(c)** Mean cortical surface area (SA). **(d)** Mean local gyrification index (LGI).

The CATS dataset also displayed biological patterns consistent with known developmental and sex-related differences, further supporting its quality. ICV remained stable across the studied age range (Fig. 5a), while CTh, SA, and LGI decreased with age, mirroring established developmental trajectories^76,77^ (Fig. 5b-d). Additionally, sex differences in sMRI measures aligned with known patterns^76,77^, with males exhibiting larger values typically associated with larger head sizes (Fig. 5).

### Non-imaging phenotyping data

**Fig. 6:**
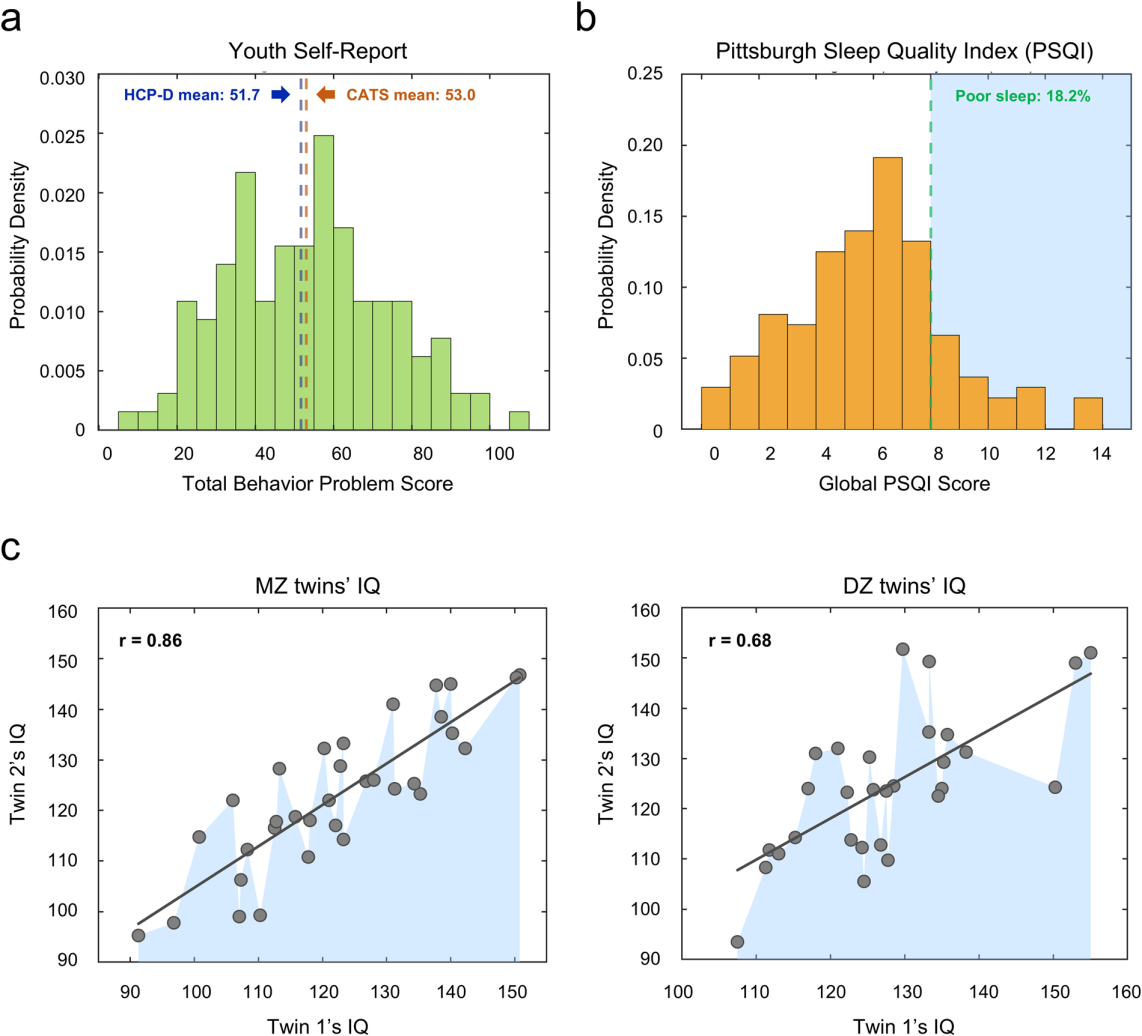
Technical validation of non-imaging phenotypes in the Chongqing Adolescent Twin Study (CATS) dataset. (**a**) Distribution of Total Behavior Problem Scores (TBPS): This panel displays the distribution of TBPS from the Youth Self-Report questionnaire (the adolescent version of the Child Behavior Checklist) within the CATS dataset. For comparison with the Lifespan Human Connectome Project Development (HCP-D) dataset, the red dashed line indicates the mean TBPS of the CATS dataset (mean = 53.0), while the blue dashed line represents the mean TBPS of the HCP-D dataset (mean = 51.7). (**b**) Distribution of Global Pittsburgh Sleep Quality Index (PSQI) Scores: This panel illustrates the distribution of global PSQI scores in the CATS dataset. A PSQI score above 7 is classified as indicative of poor sleep quality, which accounts for 18.2% of the subjects in the CATS dataset. **(c)** Scatter plots of Full-Scale Intelligence Quotient (IQ): This panel presents scatter plots of IQ scores obtained from the Chinese revision of the Wechsler Intelligence Scale for Children (C-WISC) questionnaire. The left plot corresponds to monozygotic (MZ) twins, and the right plot corresponds to dizygotic (DZ) twins, highlighting the relationship between IQ scores within twin pairs.

The CATS non-imaging data were collected from 18 standardized questionnaires (Table 2), totaling 673 items. With a low overall missing data rate of 7.42%, the dataset reflects excellent participant compliance. To assess data quality, we examined several representative phenotypes. The total behavior problem scores (TBPS) from the Youth Self-Report questionnaire (i.e., the adolescent version of CBCL)^29,78^, consisting of 112 items rated over the past six months, averaged 53.0 (SD = 20.4), closely matching the HCP-D sample (the HCP-D mean TBPS ± SD = 51.7 ± 17.3) of a similar age group, indicating high reliability and comparability (Fig. 6a). Sleep quality, assessed via the global PSQI scores, showed that 18.2% (24/132) of participants reported poor sleep^79^. This rate aligns with existing data on Chinese adolescents and further validates the dataset^80–82^ (Fig. 6b). Additionally, IQ assessments from the C-WISC showed stronger similarities among MZ twins compared to DZ twins (Fig. 6c), consistent with known genetic influences on IQ^83^. Collectively, these comparisons against the established dataset and literature confirm the robustness and reliability of the CATS non-imaging phenotyping data.

## Usage Notes

To protect the confidentiality and privacy of adolescent participants, access to the CATS dataset is governed by a data use agreement (DUA)^84^. Prospective users should complete the following steps:

1. Create an account on the Brain Science Data Center website of the Chinese Academy of Sciences (https://www.braindatacenter.cn/).
2. Download, sign, and countersign the DUA. The lead recipient (typically the principal investigator) must sign the form and obtain a countersignature from an authorized institutional official. Email the signed DUA to the corresponding author of this manuscript.
3. Submit an online access request. On the dataset’s repository webpage^73^, click “Apply for access” in the File Downloads box. In the *Describe your request* field, please include a brief statement confirming that the signed DUA has been emailed to the corresponding author.

Access is typically granted within one week of fulfilling these requirements. All data recipients are expected to adhere to the terms specified in the DUA.

## Supplementary information

Supplementary Information

## Data Availability

The scripts used in this study are available online (https://github.com/layerConnectome/CATS).
