# Supplementary material for: The Chongqing Adolescent Twin Study: An Integrative Multimodal Brain Imaging and Non-imaging Dataset
Source: Sci Data. 2025 Jul 14;12:1211. doi: 10.1038/s41597-025-05449-z (PMC12260051; doi:10.1038/s41597-025-05449-z)
Supplement: Supplementary file 1 — Supplementary Information [file 41597_2025_5449_MOESM1_ESM.pdf]

Supplementary Information for

The Chongqing Adolescent Twin Study: An Integrative Multimodal Brain Imaging and Non-imaging Dataset

Yanting Zhu[1†], Yixiao Fu[2,3†], Jiajia Han[4], Ruoming Wang[1], Xingshun Ma[5], Xiaomei Hu[6], Tao Li[7], Zhiwei Ma[1,8,9*]

Affiliations

1. School of Biomedical Engineering, ShanghaiTech University, Shanghai 201210, China
2. Department of Psychiatry, The First Affiliated Hospital of Chongqing Medical University, Chongqing 400016, China
3. Key Laboratory of Major Brain Disease and Aging Research (Ministry of Education), Chongqing Medical University, Chongqing 400016, China
4. Precision Research Center for Refractory Diseases, Shanghai General Hospital, Shanghai Jiao Tong University School of Medicine, Shanghai 201620, China
5. Department of Neurology, The First Hospital of Yulin, Yulin, Shaanxi 719000, China
6. Department of Abdominal Oncology, The Affiliated Hospital of Zunyi Medical University, Zunyi, Guizhou 563003, China
7. Affiliated Mental Health Center and Hangzhou Seventh People's Hospital, Zhejiang University School of Medicine, Hangzhou, Zhejiang 310013, China
8. State Key Laboratory of Advanced Medical Materials and Devices, ShanghaiTech University, Shanghai 201210, China
9. Shanghai Clinical Research and Trial Center, Shanghai 201210, China

†: Yanting Zhu and Yixiao Fu contributed equally to this work.

*Corresponding author: Zhiwei Ma (zhiwei.ma@shanghaitech.edu.cn)

This file includes:

Figure S1 to S9: page 2 – 10

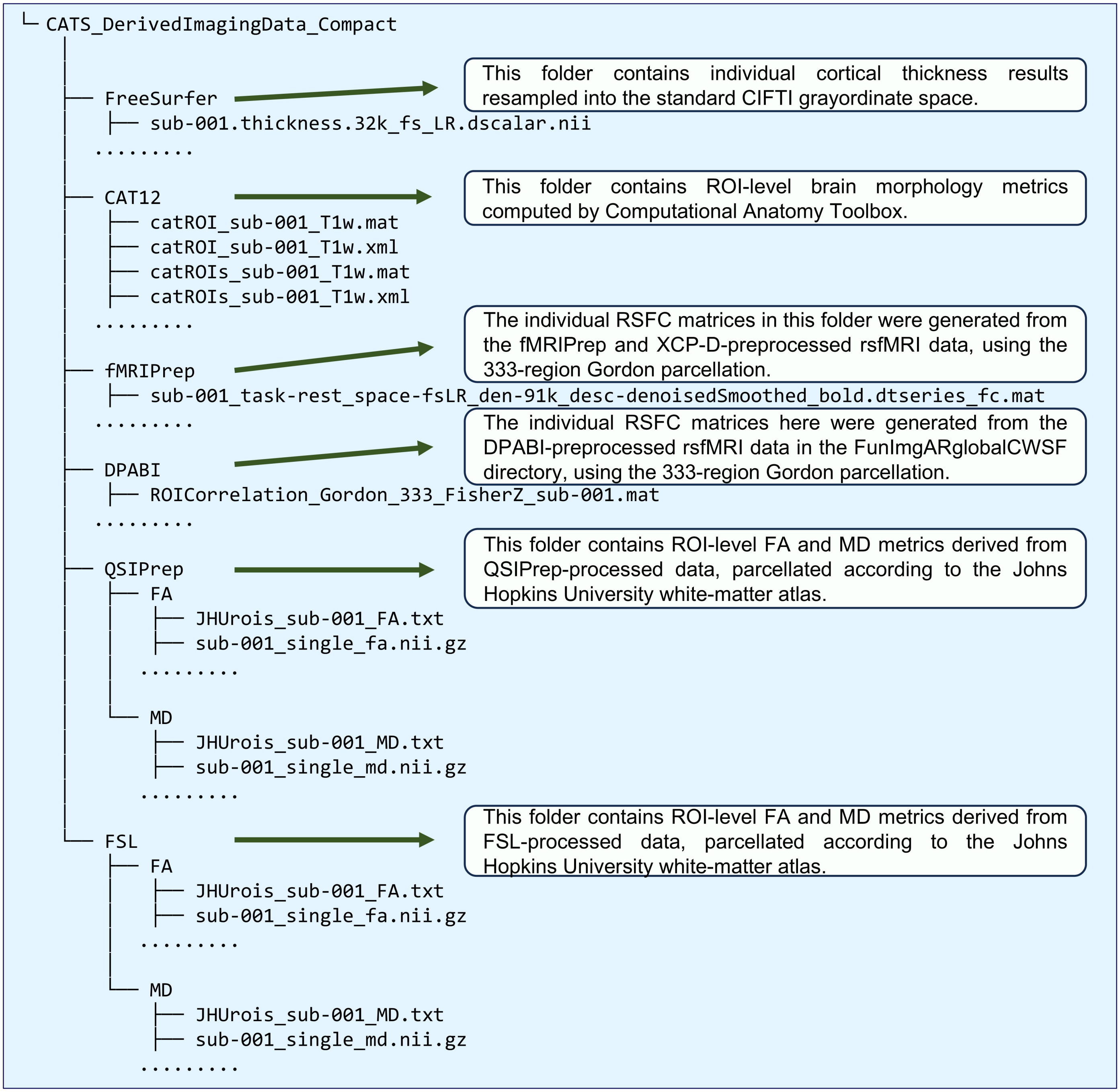

Figure S1. Contents of *CATS_DerivedImagingData_Compact.zip*, the slimmed-down version of derived imaging data. This compressed archive contains the key subject-level imaging phenotype files for every modality in the Chongqing Adolescent Twin Study (CATS). It is only 545 MB, making it quick to download.

```
└ MRIQC
    ├── mriqc_bold
    │   ├── .bidsignore
    │   ├── dataset_description.json
    │   ├── group_bold.html                      →  Group QC reports for rsfMRI data
    │   │
    │   ├── sub-001
    │   │   └── func
    │   │       └── sub-001_task-rest_bold.json
    │   ├── sub-001_task-rest_bold.html  →  Individual QC report for rsfMRI data
    │   │
    │   ├── sub-002
    │  .........
    │
    └── mriqc_t1w
        ├── .bidsignore
        ├── dataset_description.json
        ├── group_T1w.html                       →  Group QC report for sMRI data
        │
        ├── sub-001
        │   └── anat
        │       └── sub-001_T1w.json
        │
        ├── sub-002
       .........
```

Figure S2. Contents of *MRIQC.zip* (MRIQC outputs). This compressed archive contains image quality control results for the Chongqing Adolescent Twin Study (CATS) structural MRI (sMRI) and resting-state functional MRI (rsfMRI) data.

```
└ FreeSurfer
    ├── CIFTI
    │   ├── sub-001
    │   │   ├── MNINonLinear
    │   │   │   ├── Native
    │   │   │   │   ├── MSMSulc
    │   │   │   │   ├── sub-001.thickness.native.dscalar.nii
    │   │   │   │  .........
    │   │   │   │
    │   │   │   ├── ROIs
    │   │   │   │
    │   │   │   ├── xfms
    │   │   │   │
    │   │   │   ├── fsaverage_LR32k
    │   │   │   │   ├── sub-001.thickness.32k_fs_LR.dscalar.nii
    │   │   │   │  .........
    │   │   │   │
    │   │   │   ├── sub-001.164k_fs_LR.wb.spec
    │   │   │  .........
    │   │   │
    │   │   └── T1w
    │   │       ├── aparc.a2009s+aseg.nii.gz
    │   │       ├── Native
    │   │       │   ├── sub-001.native.wb.spec
    │   │       │  .........
    │   │      .........
    │   │
    │   ├── sub-002
    │  .........
    │
    └── T1w
        ├── sub-001
        │   ├── label
        │   │   ├── aparc.annot.a2009s.ctab
        │   │  .........
        │   │
        │   ├── mri
        │   │   ├── aparc.a2009s+aseg.mgz
        │   │  .........
        │   │
        │   ├── stats
        │   │   ├── aseg.stats
        │   │   ├── lh.aparc.a2009s.stats
        │   │   ├── lh.aparc.a2009s_lgi.stats
        │   │   ├── rh.aparc.a2009s.stats
        │   │   ├── rh.aparc.a2009s_lgi.stats
        │   │  .........
        │   │
        │   ├── surf
        │   │   ├── lh.area
        │   │   ├── rh.area
        │   │  .........
        │   │
        │   ├── touch
        │   │   ├── aparc.a2009s2aseg.touch
        │   │  .........
        │
        ├── sub-002
       .........
```

Standard output of Ciftify

Standard output of FreeSurfer

Figure S3. Contents of *FreeSurfer.zip* (FreeSurfer outputs). The T1w subfolder contains the FreeSurfer outputs of the Chongqing Adolescent Twin Study (CATS) structural MRI (sMRI) data in native space, whereas the CIFTI subfolder stores outputs resampled to the standard CIFTI grayordinate space.

```
└ CAT12
   ├── sub-001
   │    ├── catROI_sub-001_T1w.mat
   │    ├── catROIs_sub-001_T1w.mat
   │    ├── catreport_sub-001_T1w.pdf
   │    ├── lh.central.sub-001_T1w.gii
   │    ├── lh.pbt.sub-001_T1w
   │    ├── lh.pial.sub-001_T1w.gii
   │    ├── lh.sphere.reg.sub-001_T1w.gii
   │    ├── lh.sphere.sub-001_T1w.gii
   │    ├── lh.thickness.sub-001_T1w
   │    ├── lh.white.sub-001_T1w.gii
   │    ├── mwp1sub-001_T1w.nii
   │    ├── p0sub-001_T1w.nii
   │    ├── rh.central.sub-001_T1w.gii
   │    ├── rh.pbt.sub-001_T1w
   │    ├── rh.pial.sub-001_T1w.gii
   │    ├── rh.sphere.reg.sub-001_T1w.gii
   │    ├── rh.sphere.sub-001_T1w.gii
   │    ├── rh.thickness.sub-001_T1w
   │    ├── rh.white.sub-001_T1w.gii
   │    └── wmsub-001_T1w.nii
   │
   ├── sub-002
 .........
```

Standard output of Computational Anatomy Toolbox

Figure S4. Contents of *CAT12.zip* (Computational Anatomy Toolbox outputs). All outputs are generated from the Chongqing Adolescent Twin Study (CATS) structural MRI (sMRI) data.

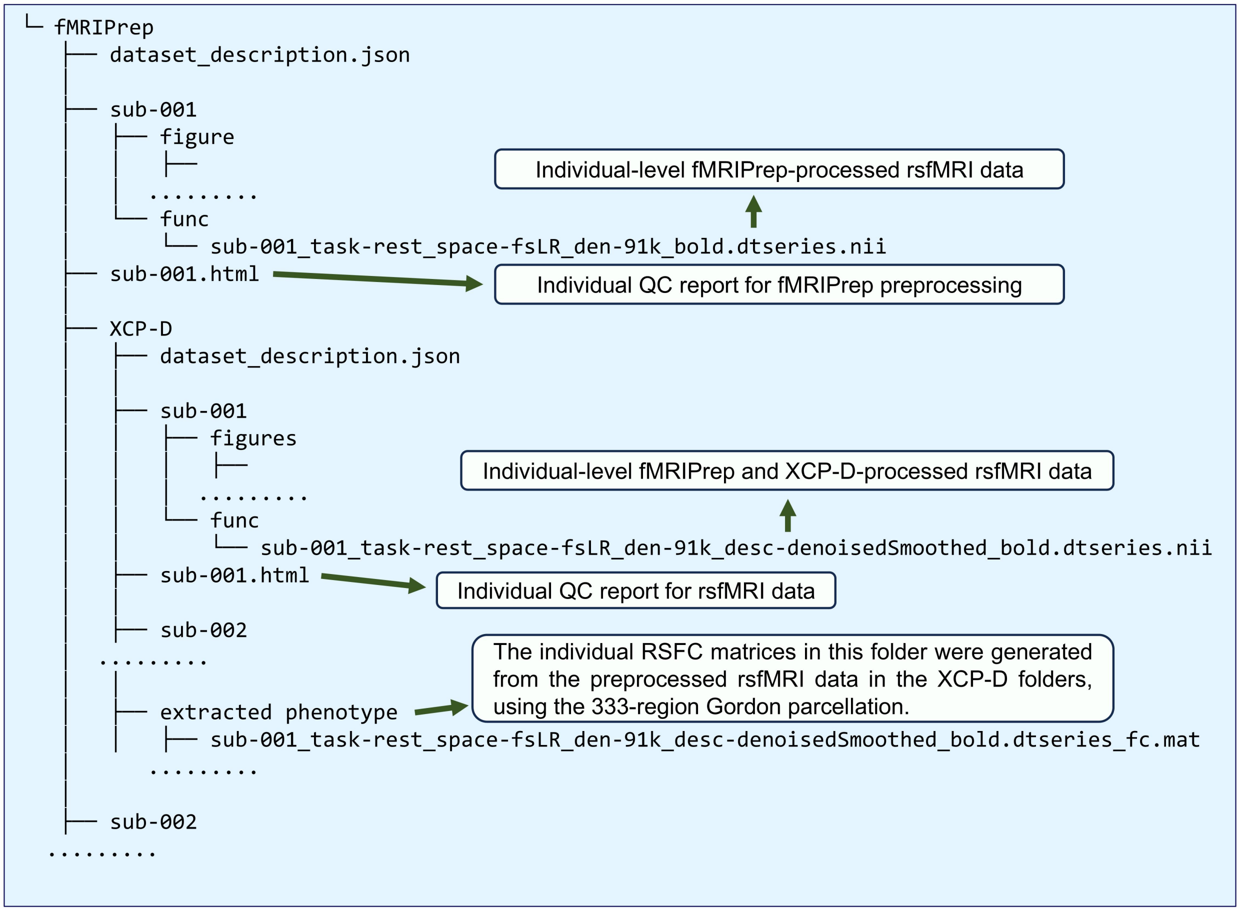

Figure S5. Contents of *fMRIPrep.zip* (fMRIPrep and XCP-D outputs). This folder contains the Chongqing Adolescent Twin Study (CATS) resting-state functional MRI (rsfMRI) data that have been pre-processed with fMRIPrep and XCP-D.

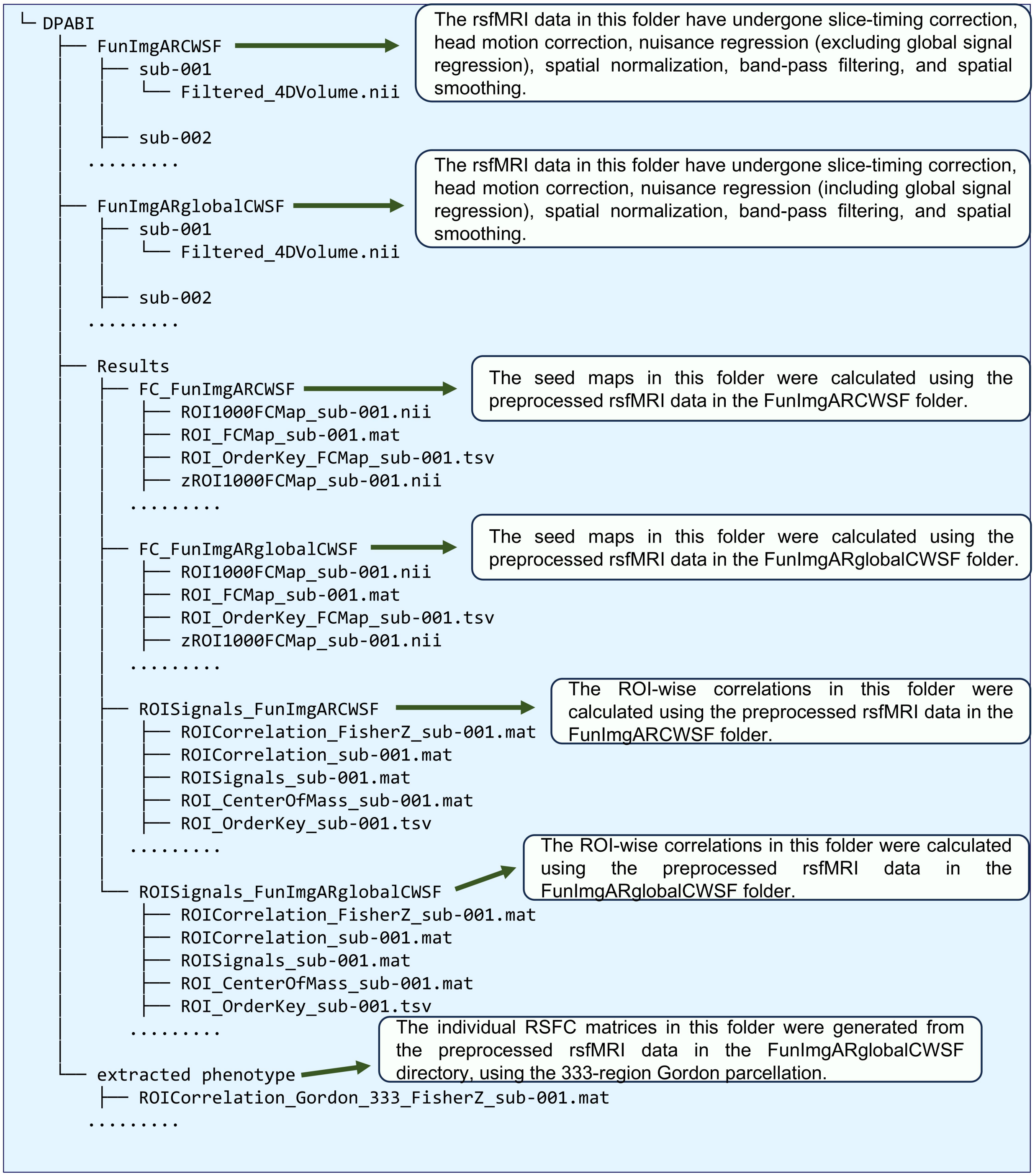

Figure S6. Contents of *DPABI.zip* (DPABI outputs). This folder contains the Chongqing Adolescent Twin Study (CATS) resting-state functional MRI (rsfMRI) data that have been pre-processed with DPABI.

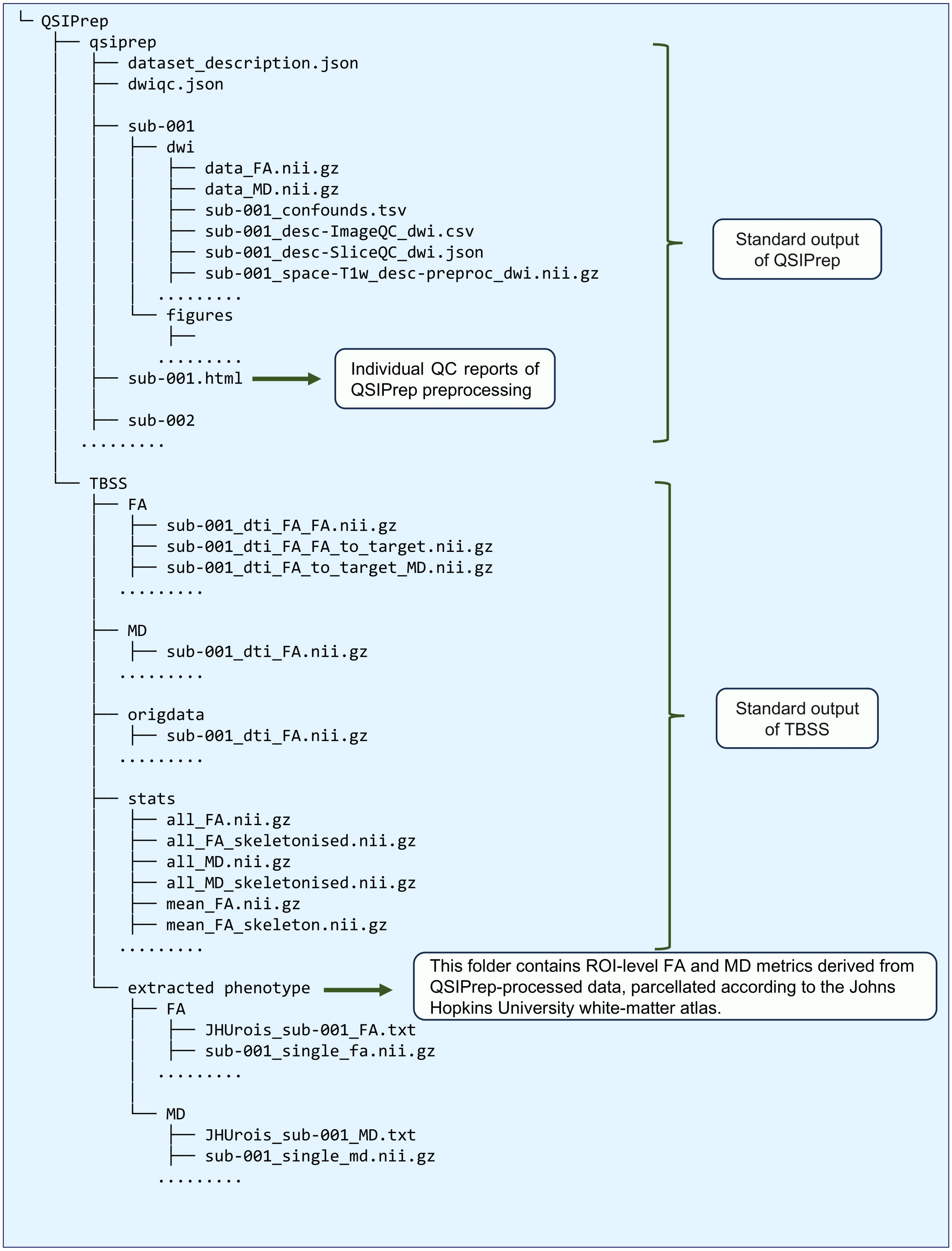

Figure S7. Contents of *QSIPrep.zip* (QSIPrep outputs). This folder contains the Chongqing Adolescent Twin Study (CATS) diffusion MRI (dMRI) quality control results and outputs generated with QSIPrep, Scilpy, and Tract-Based Spatial Statistics (TBSS).

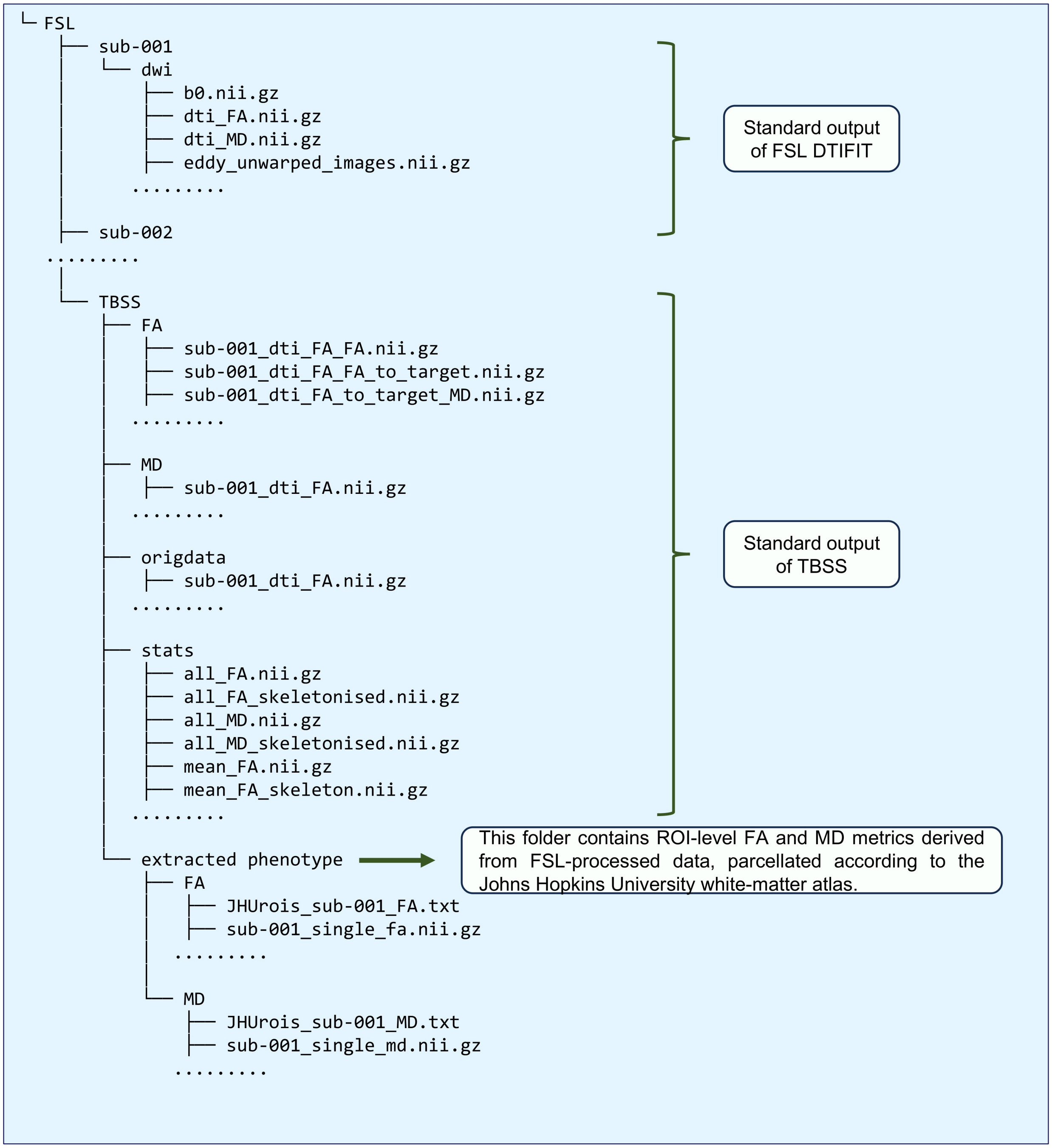

Figure S8. Contents of *FSL.zip* (FSL outputs). This folder contains the Chongqing Adolescent Twin Study (CATS) diffusion MRI (dMRI) data preprocessed with FSL tools and the resulting Tract-Based Spatial Statistics (TBSS) outputs.

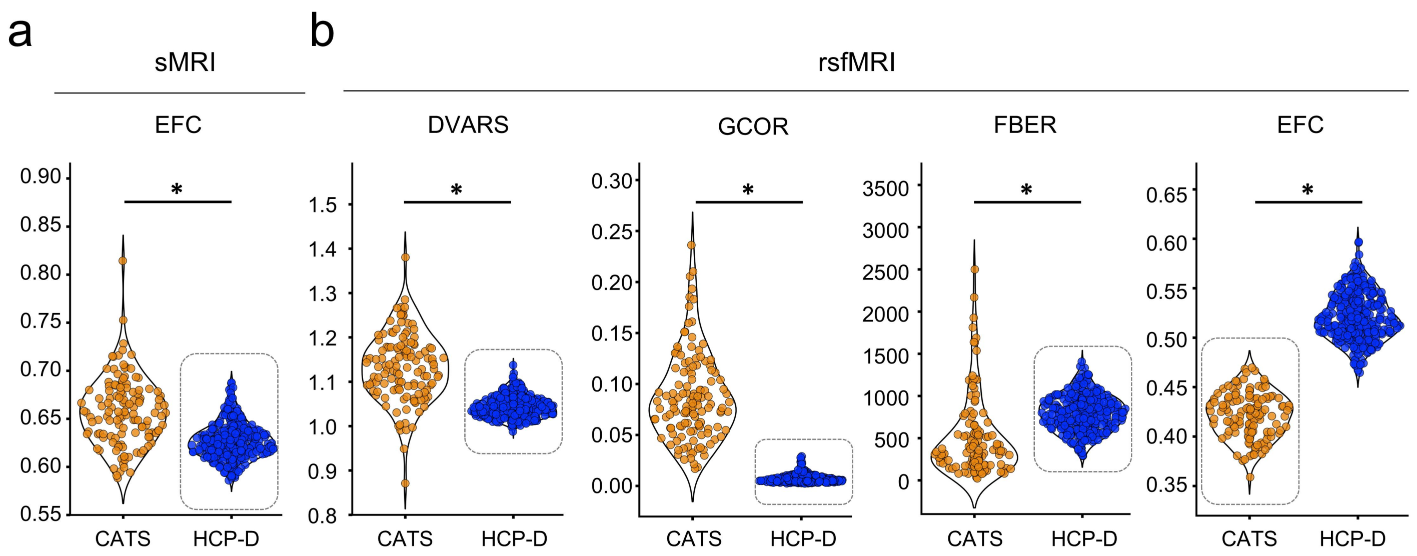

Figure S9. Additional data quality comparison between the Chongqing Adolescent Twin Study (CATS) and Lifespan Human Connectome Project Development (HCP-D) datasets. This figure presents additional data quality metrics comparing the CATS dataset with the HCP-D dataset. For each quality control metric, the beeswarm plot circled with a dashed box highlights the dataset demonstrating superior quality. An asterisk (*) indicates that the means of the two distributions are significantly different ($p < 0.05$). **(a)** Structural MRI (sMRI) quality control metrics: Entropy-focus criterion (EFC). **(b)** Resting-state functional MRI (rsfMRI) quality control metrics: Derivatives of variance (DVARS), global correlation (GCOR), foreground-background energy ratio (FBER), and EFC.
